# Effects of Vernix Caseosa on Cesarean Skin Incision: A Double-Blind Study

**DOI:** 10.3390/jcm14051527

**Published:** 2025-02-25

**Authors:** Mehmet Alican Sapmaz, Murat Polat, Sait Erbey, Omer Osman Eroglu, Inci Kahyaoglu

**Affiliations:** Obstetrics and Gynecology Clinic, Etlik City Hospital, 06170 Ankara, Turkey; dr.muratpolat@hotmail.com (M.P.); saiterbey@gmail.com (S.E.); omereroglu_94@hotmail.com (O.O.E.); mdincikahyaoglu@gmail.com (I.K.)

**Keywords:** vernix caseosa, wound, cesarean section, scar, healing

## Abstract

**Background/Objectives:** Vernix caseosa is a natural protective barrier with moisturizing, wound healing, and antioxidant properties. This study aimed to evaluate the effects of vernix caseosa on skin pigmentation and wound healing in cesarean section incisions. **Methods:** This randomized, double-blind, placebo-controlled trial was conducted at Ankara Etlik City Hospital, Gynecology and Obstetrics Clinic. A total of 210 first-time cesarean section patients were included. Vernix caseosa, collected from newborns, was applied to the incision site in half of the participants, while the other half received a placebo. Scar assessment was performed using the Manchester Scar Scale and the Patient and Observer Scar Assessment Scale (POSAS). **Results:** The vernix group showed significantly lower Manchester Scar Scale scores (9.00 ± 2.07 vs. 12.60 ± 2.72, *p* < 0.001) and POSAS total scores (53.57 ± 20.48 vs. 89.60 ± 26.54, *p* < 0.001) compared to the control group. These results suggest that vernix caseosa accelerates wound healing and improves cosmetic outcomes. **Conclusions:** Vernix caseosa emerges as a natural, effective, and low-cost method to promote wound healing in cesarean section incisions. Its medical and cosmetic benefits support its use in postoperative care. Future studies could further validate these findings in broader populations and explore additional clinical applications.

## 1. Introduction

Vernix caseosa is a natural protective barrier that begins to form physiologically in the last months of pregnancy and surrounds the fetal skin. At the same time, it has the feature of facilitating birth with its slippery structure. Vernix is thought to have a moisturizing, protective from infections, wound healing, and antioxidant role on the skin. Vernix caseosa, a unique white barrier found on the skin of newborns that resembles wax [1], is characterized by its exceptional richness in saturated branched-chain fatty acids and squalene [1,2]. Many studies have investigated whether vernix caseosa, which has a protective effect on the body of newborns, could also be effective in protecting the skin of adults [2,3]. In a study on the prevention of nipple problems developing in the early postnatal period, it was observed that vernix caseosa application was more effective and more successful in the satisfaction of mothers than breast milk application [4]. In burn injuries, skin integrity is compromised. Emerging research suggests that vernix applied to skin cultures may enhance wound healing [5]. One of the complications of a caesarean section is fetal skin laceration during the uterotomy. In a related study, it was reported that vernix caseosa applied on the laceration area of the fetal skin provided excellent healing in the early period [6]. In another study investigating the effects of vernix caseosa on wound healing, it was emphasized that vernix-based topical creams may be effective in the treatment of epidermal wounds [2]. The use of vernix caseosa in clinical settings continues to grow, and its application in surgical and dermatological practices is showing promise. In addition to its established role in neonatal care, emerging research suggests that it could be applied in treating a variety of skin conditions in adults, particularly in wound healing, tissue regeneration, and skin barrier repair. Given its unique composition and natural properties, vernix caseosa represents an exciting area of research in the field of dermatology and wound care.

Caesarean section is a common type of surgery worldwide. The decision to undertake a cesarean section is increasingly prevalent due to the heightened identification of risk factors, a growing incidence of multifetal pregnancies and premature deliveries, as well as advanced maternal age [7]. Beyond obstetric considerations, the cesarean section is also conducted for indications pertaining to ophthalmological, orthopedic, cardiovascular, neurological, and psychiatric conditions [8,9]. The most common procedure is the transperitoneal caesarean section, which involves a transverse lower abdominal incision through the skin, subcutaneous tissue, peritoneum, and uterus. Wound repair and scar development initiate with hemostasis, progressing through the stages of inflammation, tissue proliferation, and remodeling. This process spans up to 2 years, with the timeline influenced by the wound’s etiology. A key aspect of this healing involves the transition from type III collagen to type I collagen. The type I collagen, with its more organized and compact structure, enhances tissue strength while reducing its elasticity. Disruptions in the remodeling phase can lead to the development of hypertrophic, keloid, or atrophic scars. Women who have undergone a cesarean section face an elevated risk of forming pathological scar tissue due to hormonal fluctuations, the demands of infant care, alterations in the body’s center of gravity, and genetic predispositions. Women frequently report a range of symptoms related to scarring, including pruritus, tension, discomfort, reduced sensitivity (hypoesthesia), or heightened sensitivity (hyperesthesia) in the scar region, as well as sensations of pinpricking, burning, tingling, and stinging [7]. Scar-related symptoms can lead to both physical and psychological challenges, including discomfort and difficulties in accepting the scar and oneself. In severe cases, patients may perceive the scar as an unsightly or foreign element that impairs their overall functionality and well-being.

The Manchester Scar Scale is a clinical tool used to assess the severity of scars resulting from surgical procedures, especially in obstetrics and gynecology, such as cesarean sections or episiotomies. It evaluates scars based on five criteria: width, height, color, symmetry, and texture. Each characteristic is scored (usually 1–4), with higher scores indicating more severe scarring. The scale helps clinicians monitor healing, detect complications like keloid formation, and guide treatment decisions such as laser therapy or steroid injections. It is also useful for aesthetic evaluation and follow-up care to ensure optimal healing and cosmetic results. Sethukumar et al. found that body image and quality of life significantly influenced scar perception in thyroidectomy patients. They used the Manchester Scar Scale, highlighting it as a simple and useful tool for evaluating scar perception [10]. The Patient and Observer Scar Assessment Scale (POSAS) is a tool used to evaluate scars from both the patient’s and clinician’s perspectives. It consists of two components: the Patient Scale, which assesses subjective aspects like pain, itching, color, texture, thickness, and pliability, and the Observer Scale, which evaluates objective characteristics such as vascularity, pigmentation, thickness, relief, and pliability. Each aspect is rated on a 1–10 scale, with higher scores indicating worse scar quality. The POSAS is useful for clinical monitoring, assessing treatment effectiveness, and conducting research on scar healing. It is comprehensive, reliable, simple to use, and sensitive to changes in scar quality over time. Van de Kar et al. evaluated the Patient and Observer Scar Assessment Scale (POSAS) on linear surgical scars, demonstrating good internal consistency and reliability for both observer and patient scales. The results showed strong intraobserver reliability and good agreement between the scales, confirming POSAS as an effective tool for assessing linear scars [11]. Until the year 2000, all scales used to assess scar formation primarily focused on clinical observations and physical characteristics, often placing the emphasis on the clinician/observer. This approach created a situation where patient-centered symptoms, such as pain and itching, were not considered. In 2004, POSAS (Patient and Observer Scar Assessment Scale) was developed as a multidimensional tool that incorporates both the clinician’s observations and the patient’s subjective feedback. Unlike the MSS, POSAS comprehensively evaluates scar quality by considering not only the physical and visual characteristics of the scar but also symptoms experienced by the patient, such as pain and itching, addressing both the objective and subjective aspects of wound healing [12].

The aim of this study was to objectively assess the impact of vernix caseosa application on skin discoloration and wound healing at the caesarean incision site using the Manchester Scar Scale (MSS) and the Patient and Observer Scar Assessment Scale (POSAS). Specifically, the study sought to investigate whether the application of vernix caseosa could enhance the healing process of surgical wounds by promoting tissue regeneration and reducing pigmentation changes. The goal was to explore the potential therapeutic benefits of vernix caseosa in postoperative care by evaluating both skin pigmentation alterations and the progression of wound healing. This research aims to offer a novel approach to improving both aesthetic outcomes and wound healing in patients undergoing caesarean sections.

## 2. Material and Methods

This study was a randomized, placebo-controlled, double-blind trial conducted at the postnatal clinic of the Obstetrics and Gynecology Department at Etlik City Hospital in Ankara, Turkey. The study was initiated after approval from the relevant ethics committee on 26 June 2024 with the protocol code AEŞH-EK1-2024-0084 and was designed to include patients who underwent cesarean delivery in our hospital between July 2024 and December 2024 and then participated in postpartum clinic visits.

Our study consisted of 210 patients between 37 + 0- and 41 + 6-weeks’ gestation, first pregnancy, singleton pregnancy, and delivered by elective caesarean section. Indications for an elective caesarean section included fetal malpresentation (breech, transverse, face/frontal, etc.), expected fetal macrosomia, and maternal obstetric and medical conditions (fear of labour, defined vaginismus, cephalopelvic disproportion, and orthopaedic conditions).

Preoperatively, all patients received a single dose of intravenous cefazolin as prophylactic antibiotic therapy. No additional antibiotic administration was provided either during their hospital stay or after discharge. In the postoperative care of cesarean section incisions, non-dissolving surgical sutures, specifically prolene (to be removed on postoperative day 10), were used. The surgical site was covered with sterile dressings for the first 24 h following the procedure. After 48 h, the dressing was removed. The most important aspect of cesarean incision care was maintaining dryness at the incision site. After 24 h, patients were allowed to take standing showers; however, activities that could damage the incision site, such as scrubbing or rubbing, were advised against. The incision site was cleaned once daily with warm, soapy water. After cleaning, the area was thoroughly dried. The necessity of avoiding antiseptics like hydrogen peroxide, which may delay wound healing, was emphasized.

The patients’ skin was cleaned with povidone-iodine 3–4 min prior to the initiation of the surgery. A standardized surgical technique, the pfannenstiel incision, was utilized for all patients. In cases where subcutaneous tissue thickness exceeded 1 cm, interrupted sutures (3.0 vicryl rapide [polyglactin 910]) were used to close the subcutaneous layers. For skin closure, non-absorbable polypropylene (3.0 prolene) sutures were applied. A continuous suture technique with a curved needle was used for all patients. In the groups treated with vernix caseosa, a small amount of vernix caseosa was applied to the cesarean skin incision site, ensuring the incision line was not visible (Figures S1 and S2).

The wound was dressed with sterile gauze, following standard routine procedures, and secured in place with adhesive plaster tape. During subsequent ward visits, the dressings were removed by the attending specialists and nurses for evaluation and care.

Patients who came to retrieve their sutures on their postoperative day 10 were asked to fill in these scales by the physicians who evaluated them. Both the patients and the evaluating physicians did not know whether the caesarean section score lines had been treated with vernix caseosa. Patients who received Vernix caseosa (Figure S3) and those who did not (Figure S4), during the 10th postoperative day follow-up, are presented.

The patients included in the study were pregnant women who were over 18 years of age, had no disease that could pose a risk to pregnancy, underwent caesarean section for the first time, and had no dermatological skin disorders. Participants under 18 years of age; individuals with systemic diseases such as diabetes mellitus, hypertension, or gestational diabetes; those with multiple pregnancies; those with a history of smoking, alcohol consumption, drug use, or addiction; obese patients with a pre-pregnancy body mass index (BMI) of 30 or higher; patients exceeding the weight gain limits during pregnancy as revised by the Institute of Medicine [13]; and patients with hemoglobin levels below 11 g/dL, as defined by the World Health Organization for anemia in pregnant women [14], were excluded from the study. Additionally, patients who delivered meconium-stained amniotic fluid or underwent a cesarean section due to chorioamnionitis were also excluded. Furthermore, patients with any dermatological conditions such as dermatitis, keloids, or psoriasis were excluded.

Power analysis was performed to determine the required sample size. The minimum sample size required for each group in this study was determined to be 105, based on a 95% confidence interval, an alpha level of 0.05, and a statistical power of 99%. During the study period, a total of 473 primary caesarean sections were performed in the hospital. The number of patients who volunteered to participate in the study was 295. It was found that some of the 85 volunteers did not come to the postnatal follow-up, and some of them did not fill in the wound assessment scale. Because of these deficiencies, 210 patients were included in our study, 105 of whom were treated with vernix caseosa and 105 of whom were in the placebo-control group. Patients were unaware of whether vernix caseosa was applied to their cesarean section skin incisions. A researcher responsible for managing patient records was aware of which patients received vernix caseosa and which did not. During postoperative evaluations, the examiners conducted their assessments without knowledge of the treatment allocation, ensuring that they were blinded to whether patients had received vernix caseosa. This design was intended to maintain the integrity of the study as a controlled double-blind trial.

Evaluations were conducted using the Manchester Scar Scale (Scale 1) (Table 1), which was utilized solely by the investigators, and the Patient and Observer Scar Assessment Scale (Scale 2) (Table 2), completed by both the investigators and the patients. The Manchester Scar Scale is a scale used for the aesthetic and functional evaluation of scars. This scale assesses how prominent the scars are and their impact on quality of life, considering the appearance, texture, color changes, and elasticity of the scars. This scale is often used to monitor the treatment of scars in plastic surgery and wound healing processes. POSAS is a scale that assesses scars from physical, aesthetic, and emotional perspectives by both the patient and the observer. While the patient evaluates symptoms such as pain and itching, the observer examines the physical characteristics of the scars such as color and depth. It is used to monitor treatment effectiveness. High scores on both scales indicate poor recovery. Using both scales in this study was intended to enhance the reliability and objectivity of the results, offering a more comprehensive evaluation of the scar’s appearance and healing.

## 3. Results

No significant differences were observed between the vernix and control groups with respect to demographic characteristics. Specifically, there were no significant differences in age (28.30 ± 5.53 years vs. 28.70 ± 5.63 years, *p* = 0.298), gravida (*p* = 0.903), parity (*p* = 0.718), or abortus (*p* = 0.976) as detailed in Table 3. This suggests that the two groups were comparable in terms of basic demographic variables.

In contrast, significant differences were observed between the two groups regarding scar outcomes. The Manchester Scar Scale score for the vernix group was 9.00 ± 2.07, which was significantly lower than the control group score of 12.60 ± 2.72 (*p* < 0.001), indicating better scar appearance in the vernix group (Table 4). An example is given of the Manchester scar scale score for a patient treated with vernix caseosa (Table S1A) and the Manchester wound scale score for a patient not treated with vernix caseosa (Table S2A).

Furthermore, the POSAS Patient Score, which reflects the patient’s subjective assessment of scar quality, was 26.36 ± 10.08 in the vernix group, compared to 43.70 ± 13.13 in the control group (*p* < 0.001). This difference suggests that patients in the vernix group perceived their scars to be of better quality, with improved healing and aesthetic appearance (Table 4). A sample comparison is presented, illustrating the POSAS score of a patient who underwent vernix caseosa treatment (Table S1B) and the POSAS score of a patient who did not receive vernix caseosa treatment (Table S2B).

In line with this, the POSAS Observer Score, which evaluates scar appearance from an external perspective, also showed a significant difference. The vernix group scored 27.36 ± 10.30, while the control group scored 45.70 ± 13.30 (*p* < 0.001), further confirming the improved scar outcomes in the vernix group (Table 4).

Finally, when combining both the patient and observer assessments, the total POSAS score for the vernix group was 53.57 ± 20.48, which was significantly lower than the control group score of 89.60 ± 26.54 (*p* < 0.001). This supports the conclusion that the vernix group experienced a significantly greater improvement in scar healing, as measured by both subjective and objective assessments (Table 4).

The differences in measurements based on groups were analyzed using an independent samples *t*-test. The relationship between the group and categorical variables was analyzed using a chi-square test.

## 4. Discussion

In this study, we aimed to evaluate the effects of vernix caseosa application on the skin line following a cesarean section. The results revealed that the patients in the vernix caseosa group had significantly lower Manchester and POSAS scores. These findings suggest that the application of vernix caseosa contributes to improved healing of the cesarean scar line, leading to a faster healing process and lower scar scores.

Different studies have shown that vernix caseosa exhibits wound healing properties in human subjects, especially in the treatment of trophic ulcers in the lower extremities of adult patients [15]. The proposed mechanism of action is believed to involve the stimulation of tissue metabolism. Additionally, the regulation of the transepidermal water loss gradient is considered a crucial factor in the formation of the epidermal barrier and its subsequent regeneration [16]. In a study conducted by Merih et al., which assessed the efficacy of vernix caseosa in preventing nipple-related issues, it was found that the group receiving vernix caseosa experienced fewer nipple complications, such as pain, erythema, and fissuring, compared to the breast milk group. Furthermore, most women in the vernix caseosa group expressed satisfaction with its application [4].

Visscher and colleagues used two experimental models to represent different types of wounds. Study 1 involved creating epidermal wounds in a hybrid porcine model using low-energy laser ablation, while Study 2 created stratum corneum (SC) damage in a novel adult human model via tape stripping. The results showed that the vernix caseosa could provide more hydration to the stratum corneum compared to oil-in-water cream (OWC) or placebo and that healing was faster [2]. As a result of this study, they suggested that vernix-based topical creams may be effective in the treatment of wounds in premature infants and adults because vernix contains stratum corneum lipids, ceramides, fatty acids, and other lipids that support SC barrier repair [16,17]. Linoleic acid can improve lipid formation and barrier function in premature skin. The application of SC lipids increases the rate of barrier repair, while the type and proportion of ceramides also affect barrier properties [18].

Oudshoorn et al. demonstrated that sequential tape-stripping effectively creates models for skin barrier disruption and repair. They found that, while severely disrupted skin showed slower recovery, the application of vernix caseosa significantly enhanced barrier recovery, promoting rapid stratum corneum formation, and preventing epidermal thickening, making vernix caseosa a promising treatment for clinical use in skin repair [19]. Tansirikongkol et al. focused on developing barrier creams that mimic the water retention properties of vernix caseosa, a natural substance with moisturizing and skin barrier-repairing effects produced by sebaceous glands during late fetal development. Their study highlighted that, due to the immature epidermal barrier, premature infants are prone to water loss (TEWL), leading to skin issues and fluid–electrolyte imbalances. While petrolatum-based creams help alleviate these conditions, they are associated with an increased risk of infection in low birth-weight infants. In this context, vernix is suggested as a potential treatment for skin care in premature infants, with the study demonstrating that high water-phase emulsions can effectively replicate vernix’s water release properties and aid in skin barrier repair [20]. Visscher et al. investigated the postnatal adaptation of neonatal stratum corneum, finding that neonatal skin hydration decreases rapidly after birth, followed by an increase, indicating adaptive changes in water handling properties. They observed that the retention of vernix caseosa significantly enhances skin hydration and free amino acid levels, suggesting that vernix caseosa contributes to postnatal adaptation of the stratum corneum by providing free amino acids such as glutamic acid and histidine [21].

In a study investigating the application of vernix caseosa for fetal lacerations during cesarean sections, it was reported that vernix caseosa was applied to the infants’ laceration wounds, resulting in excellent wound healing outcomes. The authors highlighted the advantages of vernix, noting that it is a natural, cost-effective, and readily available material [6]. The mechanisms standing behind the wound healing by vernix caseosa are stimulation of tissue metabolism, high glutamine content, and regulation of the transepidermal water gradient [5]. As a result, the use of vernix caseosa taken directly from the infant’s body surface to effectively heal ruptures resulting from a cesarean section has been implemented at Stella Maris Hospital in Medan, Indonesia, for approximately 10 years [6].

This study has several limitations and shortcomings. Factors that may influence wound healing, such as the patient’s general health status, hormonal changes, genetic factors, and nutritional status, have not been sufficiently considered. Additionally, the effective application amount of vernix caseosa has not been standardized. The postoperative follow-up period is limited to only ten days, which overlooks the long-term healing process. The study includes only women undergoing their first cesarean delivery, making it difficult to generalize the findings to a broader population. Furthermore, the subjective assessment scales used are based on personal perceptions, which may introduce biases. Future studies should include larger patient populations, longer follow-up periods, and comparisons with other treatment methods to provide more comprehensive data.

In this study, we investigated the potential benefits of applying vernix caseosa to the caesarean incision site and its effect on wound healing and scar quality. Our results indicate that the application of vernix caseosa significantly improved both the healing process and aesthetic outcome of caesarean scars, as evidenced by lower Manchester and POSAS scores. This suggests that vernix caseosa plays a key role in accelerating tissue regeneration, reducing pigmentary changes, and improving overall skin recovery.

The favorable results observed in this study are consistent with previous research highlighting the moisturizing, antimicrobial, and antioxidant properties of vernix caseosa, which help to stimulate tissue metabolism and regulate the transepidermal water loss gradient. In addition, the ability of vernix caseosa to support epidermal barrier regeneration highlights its potential in postoperative care, not only in caesarean sections, but also in other surgical wound and skin repair applications.

Although this study was limited to a specific patient population and a short follow-up period, its double-blind, placebo-controlled design adds significant strength to the findings. The results suggest that vernix caseosa, as a natural, inexpensive, and readily available material, offers a promising alternative to traditional postoperative care. By improving both functional and aesthetic outcomes, it may enhance patient satisfaction and contribute to faster, more efficient healing.

Future studies with larger cohorts and longer follow-ups are needed to further validate these findings and explore the broader applicability of vernix caseosa in different surgical settings. In addition, comparisons with other treatments will provide a deeper understanding of its comparative effectiveness. Given its potential to improve wound healing and reduce scarring, vernix caseosa may prove to be a valuable tool in clinical practice, offering a natural solution to improve postoperative recovery and cosmetic outcomes.

This study paves the way for future research into the therapeutic use of vernix caseosa, particularly in minimally invasive surgery and skin regeneration treatments. By broadening the application of this novel method, we can improve patient care and contribute to the development of more efficient and cost-effective treatment options for wound healing and scar management.

## Figures and Tables

**Table 1:** Manchester Scar Scale (Scale 1).

Color	Perfect Slight mismatch Obvious mismatch Gross mismatch	1 2 3 4
Matte vs. shiny	Matte Shiny	1 2
Contour	Flush with surrounding skin Slightly proud/Indented Hypertrophic Keloid	1 2 3 4
Distortion	None Mild Moderate Severe	1 2 3 4
Texture	Normal Just palpable Firm Hard	1 2 3 4

**Table 2:** Patient and Observer Scar Assessment Scale (POSAS) (Scale 2).

	1 = normal skin	Worst scar imaginable = 10
For Observer	1 2 3 4 5	6 7 8 9 10
Vascularity		
Pigmentation		
Thickness		
Relief		
Pliability		
Surface Area		
Overall Opinion		
Patient Scale	1 = no, not at all 1 2 3 4 5	Yes, very much = 10 6 7 8 9 10
Has the scar been painful?		
Has the scar been itching?		
Is the scar color different from the color of your normal skin at present?		
Is the stiffness of the scar different from your normal skin at present?		
Is the thickness of the scar different from your normal skin at present?		
Is the scar more irregular than your normal skin at present?		
What is your overall opinion of the scar compared to normal skin?		

**Table 3:** Comparison of demographic characteristics of vernix and control group.

	Vernix Caseosa-Applied Group (*n* = 105)	Control Group (*n* = 105)	*p*-Value
Age * (Year)	28.3 ± 5.5	28.7 ± 5.6	0.298
Gravida **			0.903
1	51 (48.6%)	48 (45.2%)	
2	35 (33.3%)	40 (38.1%)	
≥3	19 (18.1%)	17 (16.2%)	
Parity **			0.718
1	55 (52.9%)	54 (51.4%)	
2	34 (32.7%)	39 (37.1%)	
≥3	15 (14.5%)	12 (11.4%)	
Abortus **			0.976
0	92 (87.6%)	93 (88.6%)	
≥1	13 (12.4%)	12 (11.5%)	

* = Independent samples *t*-test; ** = Chi-square test.

**Table 4:** Comparison of Manchester Scar Scale and POSAS score of vernix and control group.

	Vernix Caseosa-Applied Group	Control Group	*p*-Value
Manchester Scar Scale Score	9.0 ± 2.1	12.6 ± 2.7	0.000
POSAS Patient Score	26.4 ± 10.1	43.7 ± 13.1	0.000
POSAS Observer Score	27.4 ± 10.3	45.7 ± 13.3	0.000
POSAS Total Score	53.6 ± 20.5	89.6 ± 26.5	0.000

Independent samples *t*-test.

## Data Availability

All data will be shared by the researchers if requested.

## Supplementary Materials

The following supporting information can be downloaded at https://www.mdpi.com/article/10.3390/jcm14051527/s1. Figure S1: Examples of patients in whom we applied intraoperative vernix caseosa-1; Figure S2: Examples of patients in whom we applied intraoperative vernix caseosa-2; Figure S3: Intraoperative vernix caseosa application, postoperative 10th day control; Figure S4: Intraoperative vernix caseosa was not applied to the control group patient, postoperative 10th day follow-up; Table S1A: Manchester Scar Scale of a patient treated with vernix caseosa; Table S1B: POSAS of a patient treated with vernix caseosa; Table S2A: Manchester Scar Scale of a patient without Vernix caseosa application; Table S2B: POSAS of a patient without Vernix caseosa application.
