# Supplementary material for: Effects of Vernix Caseosa on Cesarean Skin Incision: A Double-Blind Study
Source: J Clin Med. 2025 Feb 25;14(5):1527. doi: 10.3390/jcm14051527 (PMC11900010; doi:10.3390/jcm14051527)
Supplement: Supplementary file 1 [file jcm-14-01527-s001.zip › jcm-3437082-supplementary.pdf]

Supplementary Materials

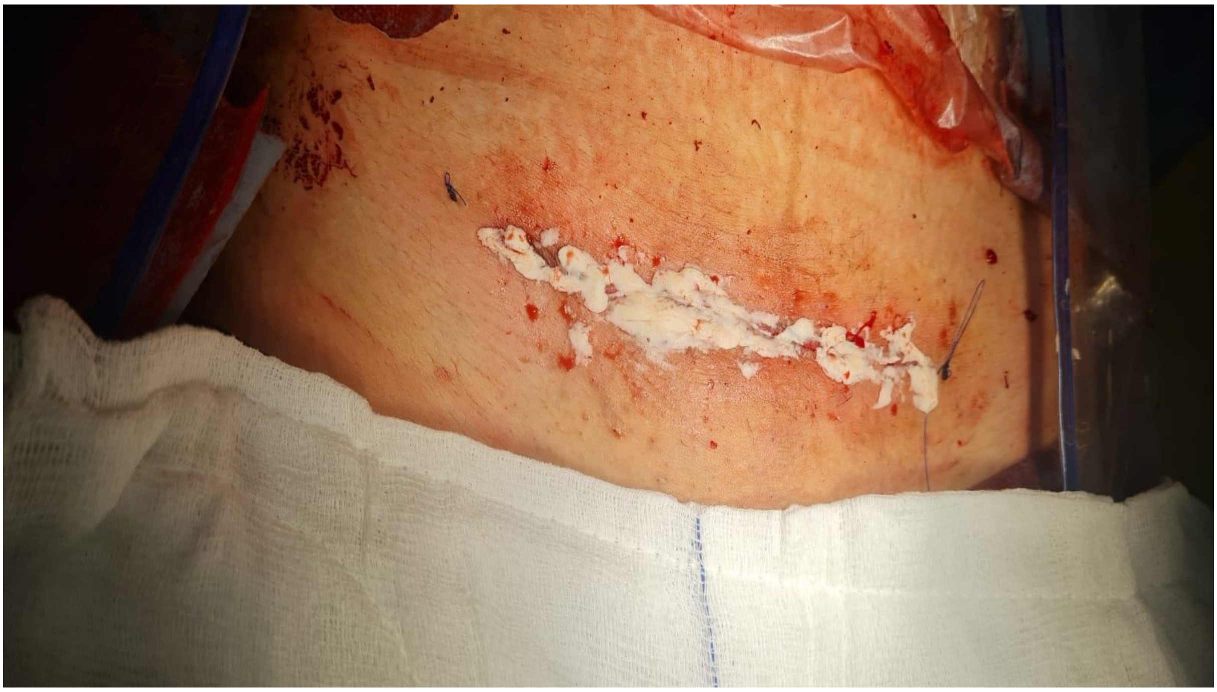

Figure S1. Examples of patients in whom we applied intraoperative vernix caseosa-1

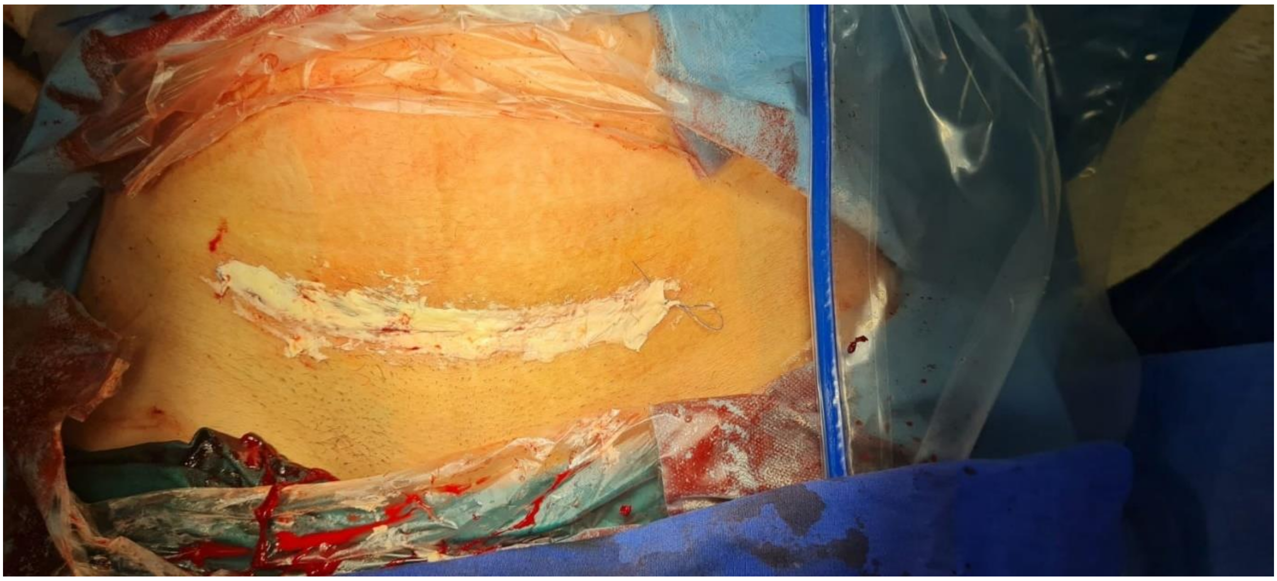

Figure S2. Examples of patients in whom we applied intraoperative vernix caseosa-2.

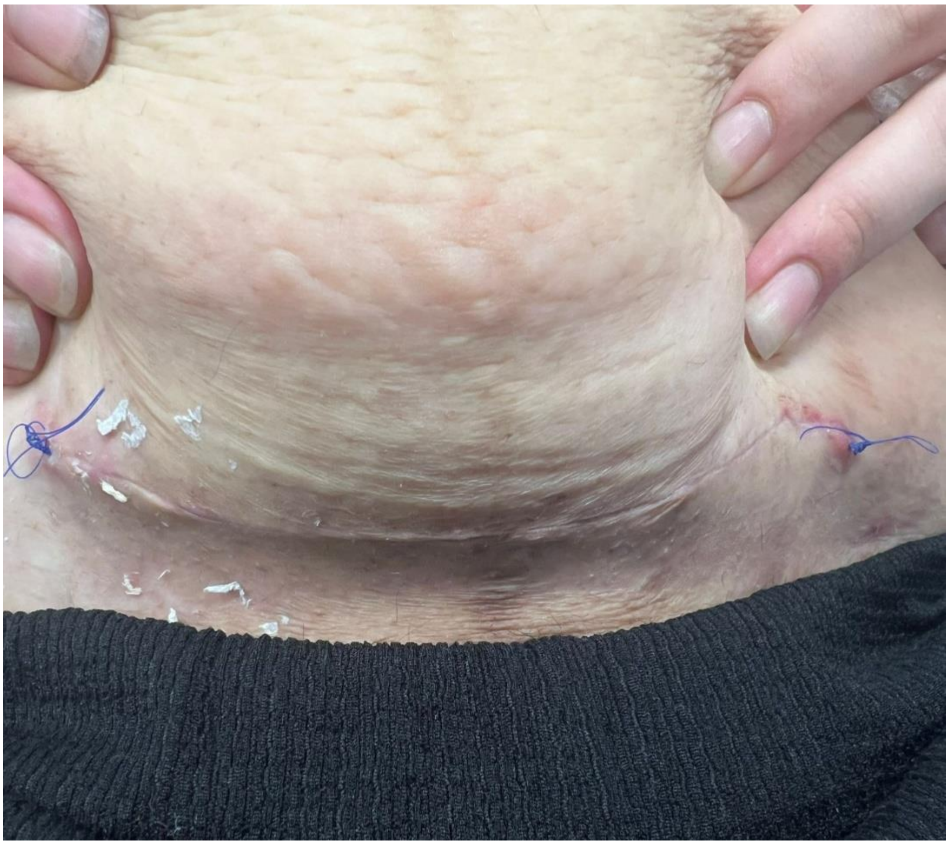

Figure S3. Intraoperative vernix caseosa application, postoperative 10th day control

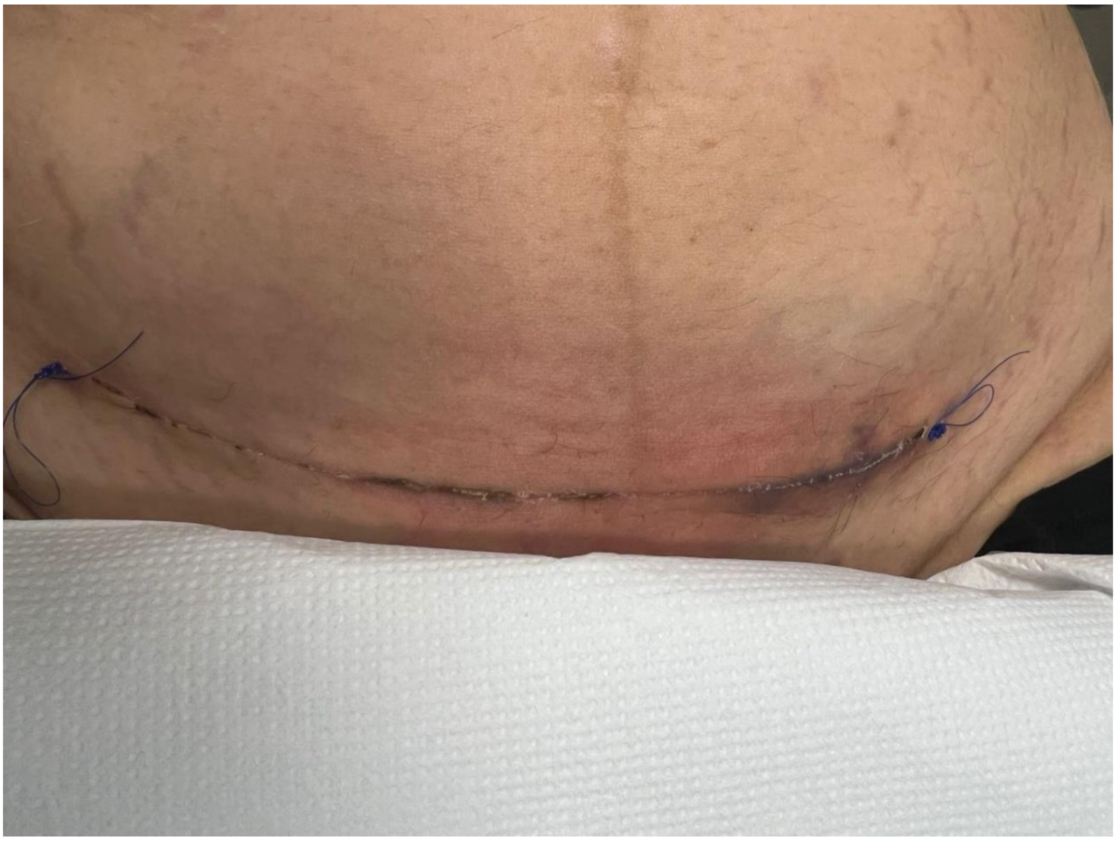

Figure S4. Intraoperative vernix caseosa was not applied to the control group patient, postoperative 10th day follow-up

Table S1A. Manchester Scar Scale of a patient treated with vernix caseosa

Color	Perfect<br>Slight mismatch<br>Obvious mismatch<br>Gross mismatch	1<br>2<br>3<br>4
Matte vs shiny	Matte<br>Shiny	1<br>2
Contour	Flush with surrounding skin<br>Slightly proud/Indented<br>Hypertrophic<br>Keloid	1<br>2<br>3<br>4
Distortion	None<br>Mild<br>Moderate<br>Severe	1<br>2<br>3<br>4
Texture	Normal<br>Just palpable<br>Firm<br>Hard	1<br>2<br>3<br>4

Manchester Scar Scale Score: 9

Table S1B. POSAS of a patient treated with vernix caseosa

	1 = normal skin	worst scar imaginable = 10
For Observer	1 2 3 4 5	6 7 8 9 10
Vascularity	2	
Pigmentation	3	
Thickness	3	
Relief	4	
Pliability	4	
Surface Area	4	
Overall Opinion	3	
Patient Scale	1 = no, not at all<br>1 2 3 4 5	yes, very much = 10<br>6 7 8 9 10
Has the scar been painful?	4	
Has the scar been itching?	5	
Is the scar color different from the color of your normal skin at present?	2	
Is the stiffness of the scar different from your normal skin at present?	4	
Is the thickness of the scar different from your normal skin at present?	3	

Is the scar more irregular than your normal skin at present?	3	
What is your overall opinion of the scar compared to normal skin?	4	

POSAS Observer Score: 23 Patient Score: 25

Table S2A. Manchester Scar Scale of a patient without Vernix caseosa application.

Color	Perfect<br>Slight mismatch<br>Obvious mismatch<br>Gross mismatch	1<br>2<br>3<br>4
Matte vs shiny	Matte<br>Shiny	1<br>2
Contour	Flush with surrounding skin<br>Slightly proud/Indented<br>Hypertrophic<br>Keloid	1<br>2<br>3<br>4
Distortion	None<br>Mild<br>Moderate<br>Severe	1<br>2<br>3<br>4
Texture	Normal<br>Just palpable<br>Firm<br>Hard	1<br>2<br>3<br>4

Manchester Scar Scale Score: 13

Table S2B. POSAS of a patient without Vernix caseosa application.

	1 = normal skin	worst scar imaginable = 10
For Observer	1 2 3 4 5	6 7 8 9 10
Vascularity	5	
Pigmentation	6	
Thickness	6	
Relief	5	
Pliability	7	
Surface Area	5	
Overall Opinion	6	
Patient Scale	1 = no, not at all<br>1 2 3 4 5	Yes, very much = 10<br>6 7 8 9 10
Has the scar been painful?	6	
Has the scar been itching?	4	

Is the scar color different from the color of your normal skin at present?	7	
Is the stiffness of the scar different from your normal skin at present?	6	
Is the thickness of the scar different from your normal skin at present?	5	
Is the scar more irregular than your normal skin at present?	8	
What is your overall opinion of the scar compared to normal skin?	7	

POSAS Observer Score: 40   Patient Score: 43
